# Analysis of risk factors and construction of predictive model for post-endoscopic retrograde cholangiopancreatography pancreatitis

**DOI:** 10.12669/pjms.39.6.7972

**Published:** 2023

**Authors:** Ping Zhu

**Affiliations:** Ping Zhu Department of Gastroenterology, The first people’s Hospital of Linping District, Hangzhou 311100, Zhejiang Province, P.R. China

**Keywords:** Endoscopic retrograde cholangiopancreatography, Pancreatitis, Risk factors, Prediction model, Nomogram

## Abstract

**Objective:**

To explore the risk factors of post-endoscopic retrograde cholangiopancreatography (ERCP) pancreatitis (PEP) and to establish a predictive model.

**Methods:**

This was a retrospective observational study. Patients diagnosed with calculous cholangitis and treated with ERCP (n=998) in The First People’s Hospital of Linping District from January 2014 to September 2022 were included. Risk factors of PEP were identified using univariate and multivariate logistic regression, and a nomograph prediction model was established based on the identified independent risk factors.

**Results:**

PEP occurred in 52 patients (5.2%). Logistic regression analysis showed that common bile duct diameter, history of PEP, operation time, intubation frequency, pancreatic ducts visualization, and Sphincter of Oddi dysfunction (SOD) were independent risk factors for inducing PEP (P<0.05). The calibration curve showed that the predicted probability of occurrence of the nomograph model was consistent with the actual probability of occurrence. The C-index value calculated by the Bootstrap method was 0.966, suggesting the nomograph prediction model has a good discrimination ability. The AUC of the nomograph prediction model ROC curve was 0.966 (95% CI: 0.857-0.941), suggesting good prediction efficiency, and the decision analysis curve shows a high value.

**Conclusions:**

Independent risk factors for PEP are large diameter of common bile duct, history of pancreatitis, long operation time, high intubation frequency, pancreatic ducts visualization, and SOD. The nomogram prediction model based on the above independent risk factors has good prediction ability.

## INTRODUCTION

In recent years, the incidence of pancreaticobiliary diseases in China has increased year by year due to congenital heredity, physical factors, living habits and social environmental factors.^1^ These diseases cause significant patient burden and decreased health and quality of life.^2^ clinical symptoms of pancreaticobiliary disease manifest as pain in the liver, gallbladder and pancreas, abdominal distension, diarrhea, and dyspepsia.^3^ If effective treatment is not taken in time, the patient’s water electrolyte and acid-base balance can easily be broken down.^2^-^4^

At present, endoscopic retrograde cholangiopancreatography (ERCP) is mainly used in the clinical diagnosis and treatment of various pancreaticobiliary diseases.^5^ While ERCP results in less surgical trauma it will inevitably bring postoperative complications. Post-ERCP pancreatitis (PEP) is one of the most common and severe complications of ERCP, which can reduce recovery, increase the treatment risk, but also endanger the life and health of patients.^5^,^6^ The incidence of PEP in different populations has been reported to be between 2.1% and 20.6%.^7^ It is very important to identify the relevant influencing factors of PEP and take targeted, preventive measures to reduce the risk of treatment and improve the prognosis of patients.^8^,^9^

Understanding the risk factors of PEP and establishing a prediction model are of great significance to prevent PEP. Therefore, this retrospective analysis was completed using the clinical data of 998 patients with calculous cholangitis who were treated with ERCP from January 2014 to September 2022.

## METHODS

This was a retrospective observational study. The records of patients with ERCP calculous cholangitis admitted to the First People’s Hospital of Linping District from January 2014 to September 2022 were collected retrospectively, Basic information on age and gender, and disease-related information such as diameter of common bile duct, history of pancreatitis, duration of operation time, intubation frequency, pancreatic ducts visualization, and history of Sphincter of Oddi dysfunction were collected.

### Ethical Approval

The ethics committee of the First People’s Hospital of Linping District approved this study (No. 2022-002, Date: 2022-10-09).

### Inclusion criteria:

- Calculous cholangitis patients undergoing ERCP.^10^
- Patients aged ≥18 years.
- Patients with normal serum amylase level before ERCP.

### Exclusion criteria:

- Patients with coagulopathy and hematopoiesis.
- Patients with severe cardio-cerebrovascular diseases.
- Patients with liver and kidney dysfunction.
- Patients with endocrine system diseases.
- Patients with missing clinical data.

Before ERCP, the blood coagulation function, blood sugar, blood lipid, serum electrolyte, kidney function, liver function, and urine amylase were routinely tested. The patient was anesthetized, and the position of the main duodenal papilla was confirmed. Duodenoscopy (JF-240, Olympus, Tokyo, Japan) was used and the contrast agent was injected using the contrast catheter. The extent of bile duct dilation and the number, location, and volume of common bile duct stones was determined, and then a duodenal sphincterotomy was performed with an arc-shaped incision knife and an appropriate method for stone removal was selected. The nasobiliary duct was retained within the common bile duct and the patient was observed for fever or other reactions. The nasobiliary duct was used for drainage, and the laparoscopic cholecystectomy was continued to establish pneumoperitoneum using the conventional three-hole method. Laparoscopy was used to identify important tissues and organs such as the common bile duct. After the completion of gallbladder dissection, electrocoagulation of the bleeding point of the gallbladder bed was completed.

### Judgment criteria for PEP:^11^

Routine blood work showed increased white blood cells with a shift in neutrophil nuclei. Increased serum pancreatic amylase which exceeded the normal value by three times, with a continued rise in serum lipase. The appearance and increase in serum ferroaluminum with a fasting blood glucose higher than 10mmol/L. Abdominal B-ultrasound showed enlargement of the pancreas and abnormal echo in and around the pancreas.^12^ The patient’s age, gender, history of pancreatitis, operation time, common bile duct diameter, intubation frequency, pancreatic ducts visualization and SOD were collected.

For logistic regression in identifying risk factors: The patient’s PEP was taken as the dependent variable, and the common bile duct diameter, history of pancreatitis, operation time, intubation frequency, pancreatic ducts visualization and SOD were assigned as the independent variables (Table-I).

**Table-I:** Assignment method of the risk factors in the logistic regression

Independent variable	Assignment method
Common bile duct (mm)	<8=0, ≥8=1
History of pancreatitis	No=0, Yes=1
Operation time (minute)	<50=0, ≥50=1
Intubation frequency (time)	<6=0, ≥6=1
Pancreatic ducts visualization	No=0, Yes=1
SOD	No=0, Yes=1

### Statistical analysis

All analyses were performed using SPSS 22.0 (SPSS Inc., Chicago, USA) and R 4.22 (RStudio Inc., Boston, USA). The counting data was expressed as a percentage and the chi-square test was used to compare the differences between groups. The Shapiro-Wilk test and histogram were used to determine the normality of the measurement data, and the normal distribution was expressed as mean ± standard deviation. A t-test was used to compare the differences between groups. The receiver operating characteristic (ROC) curve was used to evaluate the diagnostic effectiveness of the prediction model using the Bootstrap method to calculate the internal validation c-index test model. The calibration curve was used to evaluate the consistency between the predicted risk and the actual risk, and then the decision curve was used to analyze and evaluate the clinical efficacy of the risk model.

## RESULTS

A total of 998 patients, 499 males and 499 females were included in this study. The average age was 52.74±9.81 years. There were 52 patients diagnosed with PEP, with an incidence of about 5.2% (52/998), including 27 males and 25 females, with an average age of 51.4±8.67 years. Univariate analysis showed that common bile duct diameter, history of pancreatitis, operation time, intubation frequency, pancreatic ducts visualization and SOD were risk factors for PEP, with statistically significant differences (P<0.05; Table-II).

**Table-II:** PEP univariate logistic analysis.

Risk factors	PEP	Non-PEP	χ^2^/t	P
Male, n (%)	27(51.92)	472(49.89)	0.081	0.776
Age	51.4±8.67	52.82±9.87	-1.011	0.312
Common bile duct (≥ 8mm)	36(69.23)	412(43.55)	13.138	<0.001
History of pancreatitis (Yes)	35(67.31)	114(12.05)	118.496	<0.001
Operation time (≥ 50 minutes)	30(57.69)	308(32.56)	13.903	<0.001
Intubation frequency (≥six times)	33(63.46)	378(39.96)	11.241	0.001
Pancreatic ducts visualization (Yes)	35(67.31)	300(31.71)	28.006	<0.001
SOD (Yes)	34(65.38)	410(43.34)	9.699	0.002

The results of multivariate logistic analysis showed that the probability of occurrence of PEP was 2.689 times (95% CI: 1.366-5.294) when the diameter of the common bile duct was ≥ 8 mm. PEP was 13.04 times higher in patients without previous pancreatitis (95% CI: 6.786-25.051), while patients with an operation time ≥50 minutes resulted in a PEP occurrence 2.878 times compared to patients with an operation time <50 minutes (95% CI: 1.502-5.513). Intubation frequency ≥ 6 was 2.00 times more likely to result in PEP compared to an intubation frequency <6 (95% CI: 1.043-3.843). The pancreatic ducts visualization was 4.18 times higher than that of non-pancreatic ducts visualization (95% CI: 2.136-8.161). SOD was 2.07 times of non-SOD (95% CI: 1.065-4.039; Table-III).

**Table-III:** PEP multivariate logistic analysis.

Risk factors	β	SE	Wald/χ^2^	P	OR	95% CI	
Common bile duct	0.989	0.346	8.191	0.004	2.689	1.366	5.294
History of pancreatitis	2.568	0.333	59.404	<0.001	13.039	6.786	25.051
Operation time	1.057	0.332	10.159	0.001	2.878	1.502	5.513
Intubation frequency	0.694	0.333	4.353	0.037	2.002	1.043	3.843
Pancreatic ducts visualization	1.429	0.342	17.460	<0.001	4.175	2.136	8.161
SOD	0.729	0.340	4.598	0.032	2.074	1.065	4.039
Constant	-9.097	1.042	76.158	<0.001			

**Fig.1:**
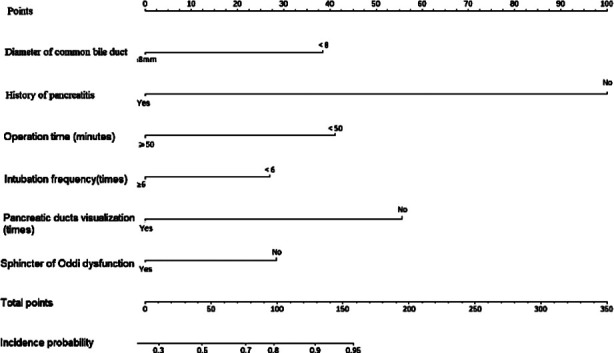
Risk model of PEP’s nomogram.

**Fig.2:**
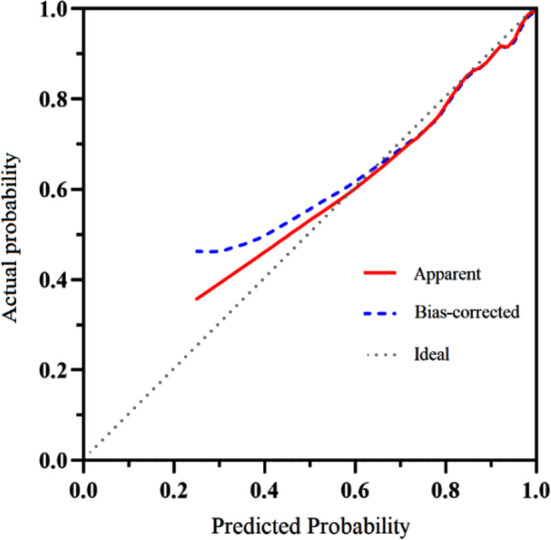
Calibration curve of nomogram model.

**Fig.3:**
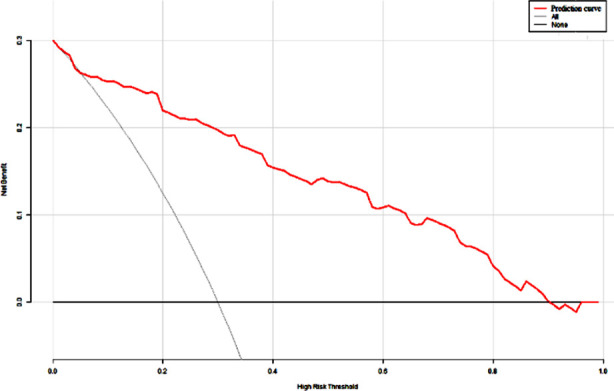
ROC curve of nomogram prediction model.

**Fig.4:**
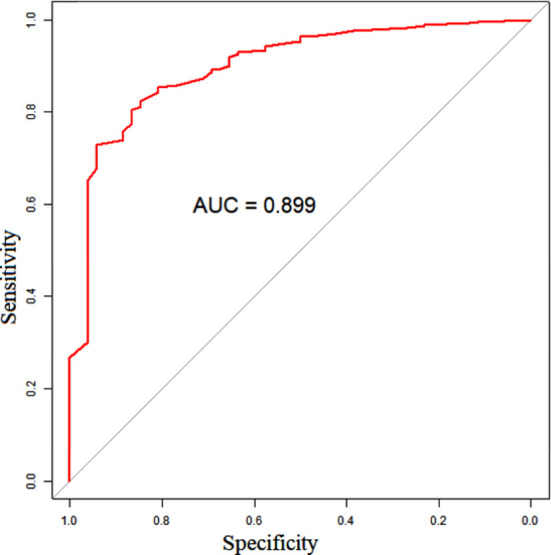
Decision curve analysis of nomogram prediction model.

Based on the independent risk factors screened by the above multivariate logistic regression analysis, R software 4.0.2 and its rms package were used to build a nomogram model of the risk of PEP and draw the Nomogram map (Fig.1). The goodness of fit of the prediction model was tested and evaluated by the Hosmer-Lemeshow method, and the results showed that χ^2^= 11.021, *P*=0.200. The internal validation of the model was carried out using bootstrap self-sampling method (sampling for 100 times). The predicted probability curve of the model was close to the actual curve, and the distribution was not far from the ideal curve, suggesting the predicted probability of PEP and the actual probability of occurrence of the model are consistent (Fig.2). The calculated C-index value was 0.966, showing the nomogram prediction model to have good distinguishing ability. The ROC curve AUC was 0.966 (95% CI: 0.857-0.941), which proves that the prediction model has good prediction efficiency and good discrimination ability (Fig.3). The decision curve analysis showed that the prediction model has high value. (Fig.4).

## DISCUSSION

The results of this study highlight that a large diameter of the common bile duct, history of pancreatitis, long operation time, high intubation frequency, pancreatic ducts visualization and SOD are all independent risk factors for PEP after ERCP. These results are consistent with a logistic analysis completed by Wang M et al.^13^ of 250 elderly patients with common bile duct stones treated by ERCP. They found that a history of SOD, gender, BMI ≥ 30 kg/m^2^, history of pancreatitis, ERCP operation time ≥60 minutes, multiple intubations, and pancreatography were all risk factors for PEP. The use of ERCP is a good surgical choice for liver, gallbladder, pancreas and other diseases, as this procedure can achieve rapid and effective infection removal while creating a minimal surgical area and avoiding massive bleeding during the operation.^5^

However, the operation time and intubation frequency can also induce secondary PEP through bacterial infection such as the degree of contact between the pancreaticobiliary duct and the outside, bile reflux, and bile duct stones.^6^,^9^ Our results correspond to work by Wang P et al.^14^ who retrospectively analyzed 440 patients treated by ERCP, 39 of whom were diagnosed with PEP, and observed an incidence of PEP of 8.86%, with Ye L et al.^15^ also observed an incidence of PEP of 7.68% (109/1419). Furthermore, a recent meta-analysis showed that the incidence rate of PEP from 1961 to 2016 increased ~3.07%.^16^

Previous work has established age, gender, SOD, past history of PEP, recurrent pancreatitis and sphincter dysfunction as risk factors for PEP post-ERCP.^17^,^18^ The results presented here are consistent with the previous work, with the exception of the association of age, gender and sphincter dysfunction. A patient’s age and gender parallel the decline of overall body function in addition to representing a decrease in protease secretion. Additionally, pancreatic cell activity in women is more prominent than in men which suggests a higher protease secretion rate.^19^,^20^

Finally, our results show that the diameter of the common bile duct and the presence or absence of pancreatic duct visualization are also independent risk factors for PEP, a conclusion which is supported by work completed by Li L et al.^21^ In general, the diameter of the common bile duct often determines the severity of disease, including the impact of pancreaticobiliary duct disease on sphincter function, operation time, and intubation frequency.^17^,^18^ As mentioned, ERCP of the larger diameter of the common bile duct has a high risk of postoperative complications. Similarly, pancreatic duct visualization is the main reference standard for imaging diagnosis of pancreaticobiliary duct disease, it is also closely related to the development and severity of disease.^22^,^23^ As such, we have shown that a large diameter of the common bile duct, history of pancreatitis, long operation time, high frequency of intubation, pancreatic duct visualization and SOD are independent risk factors for inducing PEP.^17^-^19^

Based on the identified risk factors of multivariate logistic regression analysis, this study established a nomogram model to predict the risk of PEP. The model can transform the complicated regression equation into an intuitive and easy-to-read graph, which is convenient in practical applications. Using the Hosmer-Lemeshow method, it is established that the model is accurate and valuable. This predictive model can provide a personalized evaluation of patients with PEP which allows for a baseline reference for the development of a perioperative treatment plan by quantifying the risk of PEP. Therefore, the model can not only help medical staff to develop a reasonable treatment protocol, but also prepare for postoperative complications and precautions, which can have an important impact on improving prognosis.

### Limitation of the study

This was a single-center retrospective study, and as such may have resulted in data selection or information bias. Single unit data was used for model validation, and multi-center model validation was not conducted. Additionally, a comprehensive analysis of influencing factors for patients was not included. Future analysis is needed to optimize the prediction of the model.

## CONCLUSION

A large diameter of the common bile duct, history of pancreatitis, long operation time, high intubation frequency, pancreatic ducts visualization and SOD are independent risk factors of PEP after ERCP. The nomogram prediction model based on the above independent risk factors has good prediction ability.
